# Clinical Practicum Assessment for Biomedical Science Program from Graduates’ Perspective

**DOI:** 10.3390/ijerph191912420

**Published:** 2022-09-29

**Authors:** Aarti Sharma, Taghreed Abunada, Sawsan S. Said, Rana M. Kurdi, Atiyeh M. Abdallah, Marawan Abu-Madi

**Affiliations:** 1 Department of Biomedical Sciences, College of Health Sciences, QU-Health, Qatar University, Doha 2713, Qatar; 2 Department of Public Health, College of Health Sciences, QU-Health, Qatar University, Doha 2713, Qatar

**Keywords:** clinical practicum, student satisfaction, NAACLS, biomedical science, Qatar

## Abstract

The clinical practicum for biomedical science students aims to provide graduates with the knowledge and skills required to work in diagnostic laboratory settings. This study examines graduates’ perspectives on content, teachers and clinical training and their satisfaction rates based on the skills gained during training. The study was conducted on females who graduated from Qatar University between 2015 and 2020. We used a previously validated questionnaire called CPAT-QU. Telephonic interviews were conducted and were analyzed using SPSS. The results showed a high satisfaction rate, of 80%, for the content and organization of the clinical training. The participants’ main concerns included the evaluation criteria, as 8.7% were not satisfied. The students suggested increasing the length of the training in order to obtain experience that was more practical. The students’ satisfaction with the teachers was 70% in terms of their attitude, command of knowledge and ability to convey knowledge. In total, 69.5% of the graduates claimed that their instructors were interested in teaching and 4.4% claimed their instructors conveyed disinterest in teaching. The Qatar University graduates were satisfied with the content of their clinical training. However, they reported some gaps in this training. Identifying these gaps will help in restructuring clinical training to improve student experience.

## 1. Introduction

The clinical training practicum is the key mode of learning in health professions. It is the initial career experience that consolidates practical experience and theoretical knowledge for new graduates to become proficient in the field of medical laboratory science (MLS) [1].

Graduates can transfer from education to employment through these internship programs, since quality internship programs produce contented employees and experienced workers. Additionally, according to NAACLS, these programs increase the likelihood of graduates becoming competitive through undergoing clinical training, which provides knowledge and skills that they can use in future jobs [2]. The clinical training programs serve as foundations for entry into professional work. Throughout these clinical rotation courses, graduates gain hands-on experience in a variety of laboratories. This enables them to comprehend laboratory workflows.

An effective and well-planned clinical practicum provides entry-level competencies necessary for new graduates to perform their work in the future [3,4].

The expansion of clinical laboratory jobs and duties requires highly efficient technical and managerial skills among graduates. In turn, this expansion requires MLS programs to be closely and continuously monitored, assessed and improved [5].

In an attempt to govern the quality of MLS education and professions, a set of standards were suggested by the American Society for Clinical Pathology (ASCP), the American Society of Clinical Laboratory Sciences (ASCLS), and the National Accrediting Agency for Clinical Laboratory Sciences (NAACLS) [6]. This standardized approach helped in the design of MLS programs with two basic phases: formal college education and clinical practicum [7]. During the clinical practicum, clinical experiences are completed in all areas of clinical laboratory at practical sites, under the guidance of trained and certified professionals (teachers). The clinical practicum is used to ensure that each graduate completes the minimum entry-level competencies in the cognitive, psychomotor and affective domains of learning required by the employers [8,9].

MLS programs’ improvement requires continuous outcome measurement and evaluation, which reflect the effectiveness of MLS curricula and training [5]. The best methods for assessing MLS programs include direct and indirect tools [2]. Direct tools show the knowledge of graduates through essays, quantitative tests, presentations, assignments, curriculum charts and grading systems. On the other hand, indirect tools help students to reflect on learning outcomes [9]. Indirect tools include surveys, interviews and group discussions. These may help in understanding the extent of MLS programs’ success from the students’ point of view and their feelings regarding the learning process and its environment [8,10]. Students’ efficiency at work depends on the knowledge and skills they gain during clinical training. Therefore, understanding students’ perspectives helps to modify clinical training protocols and re-define the skills derived throughout the training experience.

The MLS program offered by the Department of Biomedical Sciences, College of Health Sciences at Qatar University (QU) is a NAACLS-accredited program. It mandates the completion of the clinical practicum in the last semester of a four-year curriculum before the award of a Bachelor of Science degree in MLS. The main objective of this study was to survey MLS graduates at Qatar University using a previously developed and validated tool, called CPAT-QU, in order to gather descriptive data on students’ perceptions of the clinical practicum content, teachers and entry-level competencies required to enter their profession [11]. The study examined graduates’ perspectives on content, teachers and clinical training and their satisfaction rate based on the skills they gained during their training.

## 2. Materials and Methods

### 2.1. Sample Selection and Study Design

**Figure 1:**
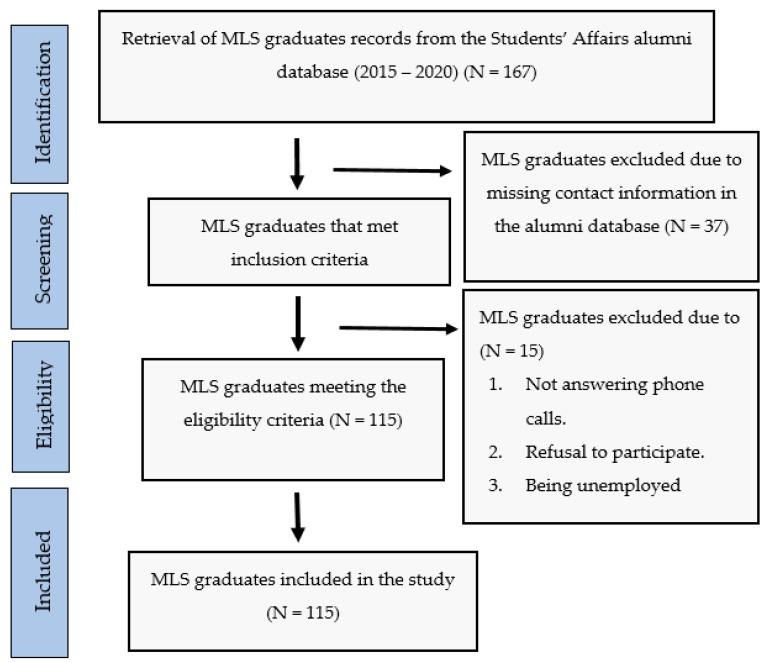
Flow chart of the study design and sampling technique.

A cross-sectional study was undertaken involving MLS graduates who successfully completed clinical practicum as part of their undergraduate program and worked in their profession at different laboratories in the state of Qatar between spring 2015 and spring 2020. The final cohort included in this study comprised 115 graduates. The study was approved by QU Institutional Review Board (Approval reference number: QU-IRB1360-EA/20) and followed all the national and international standards that apply to research with human subjects in accordance with the declaration of Helsinki. The MLS graduates voluntarily agreed to participate by electronically signing an informed-consent form. To maintain confidentiality, we assigned a code to the participants and we used these codes in the subsequent data analysis. Unemployed graduates at the time of the study were excluded. Figure 1 shows the screening process of participants. As this was a cross-sectional survey, we used Checklist for Reporting Of Survey Studies (CROSS) [12] (Table S1).

### 2.2. Research Instrument

This study made use of a previously designed and validated tool that was developed at QU. The tool is called Clinical Practicum Assessment Tool at Qatar University (CPAT-QU) and consists of 27 items (Table S2) [11]. The first part of CPAT-QU tool explores the satisfaction level of MLS graduates in terms of clinical-practicum organization, content, evaluation criteria and length. The second part of the tool inspects MLS graduates’ satisfaction with clinical teachers’ attitudes, command, ability to convey knowledge/expertise, and interest in teaching. A bipolar five-level Likert scale was used for the previous two parts of CPAT-QU tool, with answers including “very unsatisfied”, “unsatisfied”, “neutral/undecided”, “satisfied” and “very satisfied”. The third part of CPAT-QU tool examines the success in achieving entry level competencies required by new MLS graduates in the three domains of learning: cognitive, psychomotor and affective. A unipolar five-level Likert scale was used, with answers including “not at all”, “little”, “to some extent”, “well” and “very well” [13]. Entry-level-competency questions were specifically related to the clinical practicum. Questions were articulated on competencies completed in the cognitive domain of learning, such as recalling, awareness of safety and quality, awareness of laboratory operations, interpretation of laboratory results, critical analysis/problem solving and ability to retrieve/allocate information. In the psychomotor domain, questions were about graduates’ readiness to analyze samples or observe their processing, their competency and confidence in performing tasks, research skills and variable communication patterns. In the affective domain, questions were related to concepts of leadership, team-working skills, time management, initiatives, independent learning and ethical scientific behavior were explored. The CPAT-QU questionnaire concluded with an open question to assess educational needs of the new graduates.

### 2.3. Data Collection and Analysis

MLS graduates’ contact information was retrieved from the Students’ Affairs alumni database at the College of Health Sciences in September 2020. Participants were interviewed over the phone due to COVID-19 pandemic and the safety measures in the state of Qatar. The telephone interviews were administered to all participants in English. Each participant was interviewed for approximately 30 min. The interviews were not recorded due to social and cultural restrictions. The data were transferred into an Excel sheet and a descriptive analysis was carried out to obtain accurate information related to all attributes mentioned in the questionnaire. Due to the small sample number of participants, the 5-item-Likert-scale points were converted to 3-scale points for analysis.

## 3. Results

### 3.1. Characteristics of the Study Population

Out of 167 MLS graduates studying between 2015 and 2020, 130 agreed to participate in the study. One hundred and fifteen MLS graduates (88.4%) were employed in their degree profession and 15 MLS graduates had not been employed since their graduation; therefore, the final cohort included in the study was 115 graduates. Twenty-three employed graduates held a Master’s degree in Biomedical Sciences. Almost half of the graduates were working for clinical diagnostic laboratories (48.7%) and the remaining half were working in the research sector (51.3%). The work experience of the MLS graduates ranged from four months to five years at the time of the study.

### 3.2. Graduates’ Perception of Clinical Practicum

**Figure 2:**
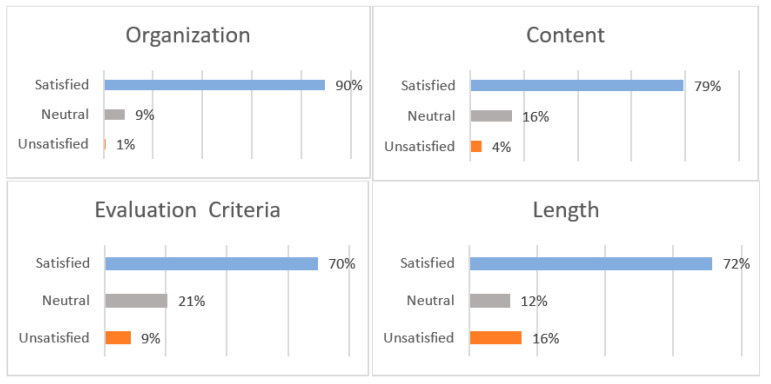
Graduates’ satisfaction rate with the clinical practicum based on four categories: (1) organization, (2) content, (3) evaluation criteria and (4) length of training.

The satisfaction rate of the MLS graduates with the clinical practicum was defined based on four categories: (1) organization, (2) content, (3) evaluation criteria and (4) length of training. The majority of the MLS graduates were satisfied with the organization (89.66%), content (79.31%), length (71.55%) and evaluation criteria (69.83%). The main concerns of the participants were related to the length of their training and the evaluation criteria during their clinical practicum. In addition, the MLS graduates suggested increasing the period of clinical training in order to obtain more practical exposure to the work environment before their graduation (Figure 2).

### 3.3. Graduates’ Perceptions of Clinical Teachers

**Figure 3:**
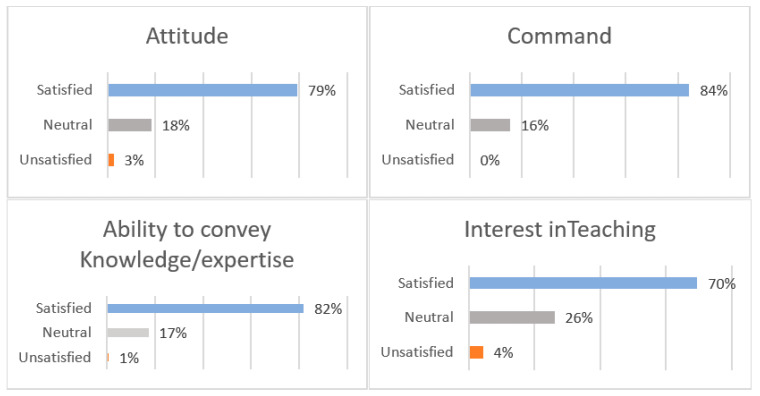
Graduates’ satisfaction rate with clinical teachers based on four categories: (1) attitude, (2) command of knowledge, (3) ability to convey knowledge and (4) interest in teaching.

The graduates gave their opinions on the clinical teachers who provided the training based on four categories: (1) attitude, (2) command of knowledge, (3) ability to convey knowledge and (4) interest in teaching. The graduates expressed good levels of satisfaction in terms of the attitudes of the clinical teachers (79.13%), as well as their command of knowledge (84.35%), ability to convey knowledge/expertise (81.74%), and interest in teaching (69.57%). The main concern reported by the graduates was dissatisfaction with some of their teachers. In total, 4.35% of the graduates reported dissatisfaction with some of their teachers due to their lack of interest in teaching (Figure 3).

### 3.4. Entry-Level Competencies

#### 3.4.1. Cognitive Domain

**Figure 4:**
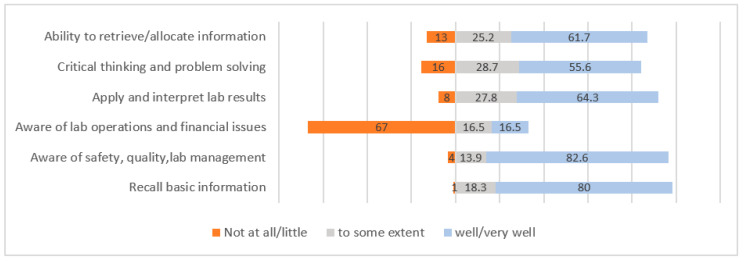
Percentages of different competencies developed in cognitive domain from graduates’ perspectives.

The results showed that the majority of the MLS graduates provided positive feedback on the competencies they developed in the cognitive domain. The graduates were efficient at applying safety and quality-control skills in laboratory settings. They were able to recall and comprehend the basic knowledge they gained during theoretical teaching. The graduates’ reported their competency in their interpretation of laboratory results, retrieval of information and critical thinking as well developed, at rates of 64.3%, 61.7% and 55.6%, respectively. Their major concerns were laboratory operations and financial skills; 67% of the MLS graduates answered with “never” or “little” (Figure 4).

#### 3.4.2. Psychomotor Domain

**Figure 5:**
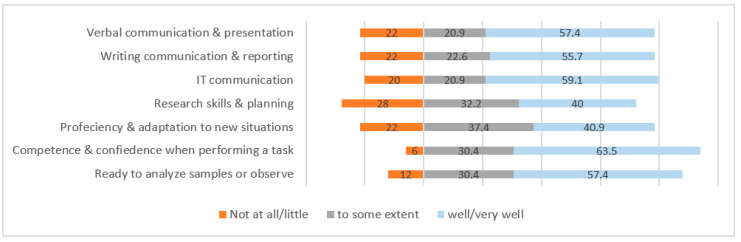
Competencies developed in psychomotor domain of learning from graduates’ perspectives.

The results showed that the participants had variable competencies in the psychomotor domain. More than half of the graduates said they were completely confident and competent in evaluating diagnostic samples, as training samples were readily available for them during clinical practicum. Proficiency and adaptation to new situations were reported as “well developed” by 40.9% and as “developed to some extent” by 37.4%. The different patterns of communication were reported to be “developed” and “developed to some extent” by 55.7% and 20.9% graduates, respectively. Verbal, written and technological communication were found to be deficient among 20–22% of the graduates. In addition, 28% of the graduates had inadequate research-related knowledge, such as how to plan and design new experiments (Figure 5).

#### 3.4.3. Affective Domain

**Figure 6:**
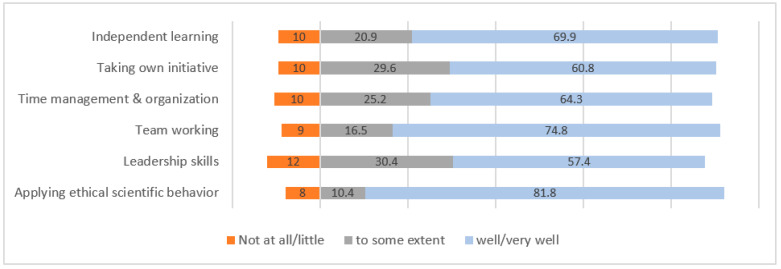
Competencies developed in affective domain of learning from graduates’ perspectives.

The graduates’ learning was well developed in ethical scientific behavior (81.8%), team working (74.8%), independent learning (69.9%), time management (64.3%), taking their own initiative (60.8%) and leadership (57.4%) (Figure 6).

## 4. Discussion

The results of this study may assist in the development of MLS programs for NAACLS accreditation and partially satisfy the NAACLS accreditation requirements for evaluation and ongoing quality improvement [2]. This survey moves beyond assessment in that it examines both the required and the sufficient elements for program improvement in accordance with assessment components laid down by the NAACLS. Additionally, these studies tend to benefit course designers, planners and policy makers while also reflecting the viewpoint of the students. This study can therefore serve as a tool to understand students’ perspectives on how to improve clinical training and also help course designers to redesign their curricula. The sample comprised biomedical students from College of Health Sciences at Qatar University. The survey demonstrated findings from interviews designed to reflect the breadth and depth of student perspectives on learning outcomes and areas of improvement related to clinical training. This research could assist the teachers and course designers by providing them with a stimulus to improve their clinical practice, as well as help in formulating improved assessment plans for students, encouraging deep thinking and fostering lifelong learning [14].

The clinical practicum provides students with an opportunity to learn basic skills and experience, as it bridges the gap between theory and practice in classrooms and the clinical environment, respectively [15]. The expansion of scientific and technological innovations in clinical laboratories induces profound changes in laboratory services, ultimately exerting a significant impact on the expected roles of new graduates. Our study showed that overall, the MLS graduates were satisfied with their clinical practicum and teachers. However, their feedback shed light on areas in which the clinical practicum may need improvement. The graduates specifically asked to increase their clinical training period and suggested modifying the evaluation criteria currently used on their MLS program [16]. A small number of the graduates were dissatisfied with their teachers’ attitudes and their lack of interest in teaching. However, these teachers were overloaded by their dual role, as they were serving as clinical educators and health care providers, with some managerial and administrative tasks at the same time. Indeed, assessing the teaching and educational practices in any health care system is crucial to maintain the quality of clinical education in any clinical professions [15,16,17,18].

Our study showed that cognitive, psychomotor and affective entry-level competencies were achieved for the majority of the graduates, which reflects the quality of the clinical practicum and program. However, there is potential for improvement in the three domains of learning. The questions related to the cognitive domain revealed that 67% of the graduates were not well versed in laboratory-management skills and financial issues. Such laboratory-management skills are vital for laboratory professionals to interpret budgets and train new professionals [4]. Furthermore, when responsibilities expand, expertise in budget control, inventories, calculation reagents, chemical consumption and purchases are required. It is recommended that laboratory practitioners improve their managerial abilities in order to advance in their careers [19].

The ability to think critically has long been regarded as a necessary skill for laboratory professionals. Employers also believe that critical thinking abilities among entry-level laboratory technologists increase patient-care quality [20]. Although critical thinking is an essential requirement in the cognitive domain, our study showed that 44.7% of the graduates did not possess this skill at all or were not confident in acquiring it. These results are similar to those of a study that showed that only 30% of pupils can use critical thinking to solve difficulties [21]. Our results also showed that clinical laboratory personnel require additional training in critical thinking [22].

The psychomotor domain of learning is critical during clinical training because it is the core of the MLS profession and aids in transferring theoretical knowledge to practical work [23]. Our findings showed that our graduates lacked research abilities related to experiment planning and design, despite the fact that 51.3% of them worked in the research sector in the state of Qatar. Other psychomotor skills that were less developed were those linked to writing reports and delivering oral presentations. The skills related to oral presentations and report writing were found to be strong among only 57.4% and 55.7% of the graduates, respectively. The lack of report-writing skills among 40.0%gradutes need to be addressed as, report writing is a mandatory in the real job market [24]. Furthermore, the research indicated that the curriculum focused on high-quality material education but missed the basic skill of training future professionals in report writing [25]. This suggests the need to develop and improve this competency among our graduates [25]. This disparity must be addressed because report writing is a prerequisite that also necessitates strong writing skills [24].

Health science is not only related to performing experiments but also includes affective and moral competencies [26]. Therefore, affective-domain skills are needed by graduates to succeed in both their personal and their professional lives. These skills include behaviors and attitudes that students need to develop throughout their clinical practice. It was gratifying to see that, in contrast to the other domains, the affective-domain skills had been acquired competently. For instance, team working is significant in increasing productivity at work and enhancing individuals’ capacity for innovative [27]. The ability to operate in a team is a skill that laboratory technicians value highly, and it was encouraging to see that 74.8% of the students developed teamwork skills during their clinical training.

Other important skills in the affective domain that are required to work in laboratory settings are time management, organization, initiative and independent learning, which were deficient among 10% of the graduates [28]. Time management is of high importance, as time sensitivity among health-care professionals leads to quality work [29]. The skills related to initiative reflect graduates’ independent-learning abilities, which are expected to be enhanced during clinical practice. The majority of our graduates agreed that they were more confident in taking the initiatives and in their independent-learning abilities were after their clinical training [30].

Our study had some limitations. For instance, it was performed by telephonic interviews due to the COVID-19 pandemic. Furthermore, the average number of graduates was only 30 per year. Although 130 graduates participated in our study, a lack of updated personal information and unemployment since graduation reduced the sample size to 115. This study was based on self-reported data that were subject to bias, as graduates might offer biased estimates of their own behavior, ranging from a misunderstanding of proper measurement to social-desirability bias, in which respondents aim to give a strong account of themselves on surveys, even if the surveys are anonymous.

## 5. Conclusions

In conclusion, this study increased the understanding of the clinical training program’s shortcomings and potential areas for future development. The study clearly showed that, although the training was well structured and covered every component of cognitive, psychomotor and affective skills, there is still room for development in the cognitive and psychomotor domains. The main area that needs improvement is the teaching of laboratory management. As encouragement, students should be given credit for the extra support they perform in laboratory management. Moreover, the students need to improve their clinical-report-writing and critical-thinking abilities. This necessitates the introduction of mandatory academic coursework. Additionally, it is important to encourage students to participate in journal-club discussions and to place more emphasis on communication skills [31]. The future reorganization of the clinical practice course with a longer training period may be necessary as a result of this additional coursework [32]. In the future, these additions may support MLS programs seeking NAACLS accreditation and partially satisfy NAACLS accreditation in terms of assessment and continuous quality improvement.

## Data Availability

Not applicable.

## Supplementary Materials

The following supporting information can be downloaded at: https://www.mdpi.com/article/10.3390/ijerph191912420/s1. Table S1: CROSS Checklist, Table S2: CPAT-QU Questionnaire.
